# Efficacy and safety of co-trimoxazole in eradication phase of melioidosis; systematic review

**DOI:** 10.1186/s12941-023-00620-z

**Published:** 2023-08-17

**Authors:** Keragala Arachchige Reshani Kaumada Keragala, Maththe Gama Ralalage Shobha Sanjeewani Gunathilaka, Rathnabahu Mudiyanselage Indika Sanjeewa Kumara Senevirathna, Jayaweera Arachchige Asela Sampath Jayaweera

**Affiliations:** 1 Department of Microbiology, Faculty of Medicine and Allied Sciences, Rajarata University of Sri Lanka, Saliyapura, 5008 Sri Lanka; 2 Department of Biochemistry, Faculty of Medicine and Allied Sciences, Rajarata University of Sri Lanka, Saliyapura, 5008 Sri Lanka

**Keywords:** Melioidosis, *Burkholderia psedomallei* infection, Co-trimoxazole, Maintenance phase, Eradication phase, Minimum safe duration, Relapses, Mortality and adverse effects

## Abstract

**Background:**

Melioidosis is an infectious disease caused by the bacterium *Burkholderia pseudomallei*. The two stages of melioidosis treatment are the intense intravenous phase and the oral eradication phase. Although co-trimoxazole has been in use for several years, the literature does not demonstrate uniformity of the drug doses, combinations, or durations suitable for the eradication phase of melioidosis. The safety profile of co-trimoxazole was not documented in the literature, nor have systematic studies of its effectiveness been done. This systematic review sought to study on the dose, duration and combination of co-trimoxazole therapy in view of clinical efficacy and safety in the eradication phase of melioidosis.

**Main body:**

This systematic review included all of the published articles that employed co-trimoxazole in the eradication phase after 1989, including, randomized clinical trials, case–control studies, cohorts, case reports, and case series. Throughout the eradication (maintenance) phase, co-trimoxazole usage was permissible in any dose for any period. A total of 40 results were included in the analysis which contained six clinical trials, one cohort study, one Cochrane review, and thirty-two case series/case reports. Clinical and microbial relapse rates are low when co-trimoxazole is used in single therapy than in combination. There were several adverse events of co-trimoxazole, however, a quantitative analysis was not conducted as the data did not include quantitative values in most studies.

**Short conclusion:**

The dose of co-trimoxazole, duration of the eradication phase, and other combinations used in the treatment was varying between studies. Compared to combined therapy patients treated with co-trimoxazole alone the mortality and relapse rates were low. The lowest relapse rate and lowest mortality rate occur when using co-trimoxazole 1920 mg twice daily. The duration of therapy varies on the focus of melioidosis and it is ranged from 2 months to one year and minimum treatment duration associated with low relapse rate is 3 months. The use of co-trimoxazole over the maintenance phase of melioidosis is associated with clinical cure but has adverse effects.

**Supplementary Information:**

The online version contains supplementary material available at 10.1186/s12941-023-00620-z.

## Background

Melioidosis is an infectious disease caused by the bacteria, *Burkholderia pseudomallei*, which can infect both humans and animals [1]. It is also called Whitmore’s disease after Whitmore and Krishnaswamy, who described the infection in 1912 [2]. Melioidosis is endemic in Northern Australia and Northeast Thailand [3, 4] while sporadically clinical cases were reported in many parts of Asia, including Myanmar, Southern India, Sri Lanka, China, Laos, Hong Kong, Mauritius, Philippines, Singapore, Indonesia, Malaysia, and Cambodia [5].

*Burkholderia pseudomallei* could be isolated commonly from contaminated soil and water, especially in the tropics [6]. *Burkholderia pseudomallei* is a Gram-negative bacillus that is oxidase positive. The ‘safety pin’ appearance was observed following Gram staining and the term bipolar staining [7]. Besides occupational exposure in farmers, the infection can be spread via inoculation, inhalation, and aspiration, especially in endemic areas [8]. The gold standard for the diagnosis of Melioidosis is *in-vitro* isolation and identification of *Burkholderia pseudomallei* in a sample of blood, urine, throat swabs, pus, or wound swabs. Even a single colony of *Burkholderia pseudomallei* is diagnostic in the symptomatic patient [7].

The incubation period of *Burkholderia pseudomallei* varies from 1–21 days, with a mean duration of 9 days [8–13]. The main clinical feature of melioidosis is fever. The course of the disease can range from acute fulminant septicemia to a localized infection, abscess formation is a characteristic feature of Melioidosis [14]. Abscesses of splenic, liver abscess, skeletal muscles, brain, prostate abscess, and parotid glands have been reported worldwide [15]. Other than that, pneumonia [16], pleural effusion [17], genitourinary infections [18], skin or soft tissue infections [19] encephalomyelitis [20], and bone or joint infections [19, 21] have been reported. Mycotic aneurysms [22], mediastinal infections [23], and thyroid and scrotal abscesses [24] have also been reported.

Treatment of melioidosis can be divided into two phases: intensive intravenous phase and oral eradication phase. In the intensive phase, ceftazidime is mainly used. Ceftazidime, 2–3 g or 40 mg/kg/dose every eight hours intravenously for 2–4 weeks is the regular dose for the melioidosis acute phase. Meropenem 1 g or 25 mg/kg every eight hours for more than two weeks is used instead of ceftazidime in severe cases [25]. However, the dosing and the duration of these drugs may vary depending on several factors such as the presence of bacteremia and co-morbid factors including diabetes, malignancies, chronic lung disease, chronic kidney diseases, thalassemia (7%), atypical mycobacterial disease, steroid therapy [26].

Oral eradication therapy, also referred to as maintenance therapy, follows the intensive intravenous treatment phase, which is the most critical phase for reducing relapses and recrudesces [27, 28]. Co-trimoxazole had been used as the drug of choice for melioidosis eradication therapy, either monotherapy or in combination with other antimicrobials. The duration of the eradication phase varies from 3 to 6 months [27, 29, 30]. When combined with doxycycline relapse rate was 4.6% while co-trimoxazole alone it was 3.2%. Compared to other bacterial infections relapses and recrudescence are common in melioidosis. Bacterial eradication is difficult and melioidosis requires prolonged antimicrobial therapy and often compliance during eradication therapy is low. Also, bacteria tend to remain within the sequestrated focus in the body and when multi-focal involvement the possibility of relapse is high. The organism leads to formation of granuloma and when host has cellular immunodeficiencies and conditions leading to secondary immunodeficiencies such as diabetes the possibility of developing relapses are high. The reason for prolonged eradication phase is to minimize the relapses and the clinical failure. Prolonged use of co-trimoxazole is associated with adverse effects like myelosuppression and skin rashes. *B. pseudomallei* is intrinsically resistant to aminoglycosides like gentamicin, amikacin, streptomycin and tobramycin, penicillin, ampicillin, first- and second-generation cephalosporins and intermediate results to quinolones. As a result, co-trimoxazole is considered as the drug choice due to the susceptibility and good tissue penetration ability. However, approximately 25% of patients with recurrent melioidosis were discovered to have new infections rather than relapses of their original infection [31, 32].

Co-trimoxazole is the combination of trimethoprim and sulfamethoxazole [20]. Although it has been used for long years, the exact dose and the required duration in the eradication phase are not uniformly available in the medical literature.

To our knowledge, this is the first systematic review which had been conducted to assess the efficacy and the safety profile of co-trimoxazole in the management of melioidosis. This systematic review aimed to analyse the evidence of co-trimoxazole for eradication therapy systematically to synthesize recommendations on the best dose, combinations, and durations of co-trimoxazole in terms of clinical efficacy and safety.

### Search strategy

We developed this search strategy following the Preferred Reporting Items for Systematic Reviews PRISMA search strategy [21].

### Inclusion criteria

All the published articles, including randomized clinical trials (RCT), case–control studies, and cohorts in which co-trimoxazole was used in the eradication phase after 1989, were eligible for this systematic review. Due to paucity, we have included case reports and case series as well. All studies were limited to human research only. The co-trimoxazole use could be in any dose for any duration during the eradication (maintenance) phase.

### Exclusion criteria

In this, we excluded the articles published in languages other than English. Also, all the studies on the pediatric population and animal studies were excluded. The search was carried out for two months, starting in May 2022. Electronic databases and grey literature were searched after finding the appropriate keywords. An electronic search of PubMed (advanced search) [22], Science Direct (Expert search) [23], Trip (PICO search) [24], Google Scholar (Advanced search) [25], Cochrane Library (Advanced search) [26] and Open-Grey [27] were done. Other than that, reference articles of the included articles were also searched for relevant articles. MeSH and other related terms were used while searching to obtain maximum coverage. We registered this systematic review in the PROSPERO (prospectively registered systematic reviews) under CRD42022345027.

### Outcome measures

The outcome measures considered in this review were: microbial failure, one-year non-relapse rate that is the appearance of clinical features of melioidosis after initial improvement, in association with cultures from any site positive for *Burkholderia pseudomallei*. The relapse can be at any period during or after stopping antibiotic treatment, clinical recurrence is the presence of recurrent clinical features of melioidosis, but not confirmed by positive culture; recurrent melioidosis is the emergence of novel signs and symptoms of infection after the onset of an oral antibiotic response and associated with a *B. pseudomallei* positive culture. Based on the typing of isolates from the first and subsequent episode, if similar is termed relapse while different considered as re-infection. Treatment failure is the clinical decision to change treatment according to inadequate response to therapy; mortality at one year and adverse drug reactions. We also calculated the mean of the duration and dose of co-trimoxazole in the eradication phase.

### Study selection

Study selection was performed by two authors independently. Both authors searched the studies on their own, downloaded the search results as.csv files, and entered them into Rayyan intelligence System separately. Then all the abstracts were screened separately. If abstracts were unclear, details were not available, or no abstracts were available full articles were reviewed. Based on exclusion and inclusion criteria, articles were categorized as excluded, included, and doubtful articles were labelled as 'may be’. Further discussions with the involvement of the supervisory author, resolved conflicts between the selections by the two authors.

### Data extraction

The data were extracted separately for each type of study. The controlled trials, cohort, and case–control studies and case reports and case series were included. Usually, for systematic reviews only research articles and reviews are included, herein, due to a low number of such articles, we have included case reports to ascertain more data on adverse effects and valuable clinical findings with dosage.

Data extracted from studies include the year of publication, site of infection, drug combinations given in the eradication phase of the disease, co-trimoxazole dose, the duration, cumulative dose, primary outcome, mortality rate, reported side effects and the methods used in monitoring were extracted.

### Quality assessment

Quality assessment was done using the Cochrane Risk of Bias assessment tool (Additional file 1: Table S1), the NIH quality assessment tool for observational cohort and cross-sectional studies (Table 1), and the NIH quality assessment tool for case series (Additional file 2: Table S2).

**Table 1:** Summary of case reports where co-trimoxazole is used as eradication therapy

Study	Study design	Site of infection	Drugs used in eradication phase	Dose	Duration	Side effects	Outcomes
Saravu *et. al*. [40]	Case series	Case 1—liver, blood, lungs		Not given	Not given		
		Case 2- liver, spleen, lungs, bones	Oral co-amoxiclav and Oral Co-trimoxazole	Not given	24 weeks	Weight gain	Splenomegaly completely regressed; hip pain subsided
		Case 3-liver, spleen		Not given	Not given		
		Case 4- liver, spleen, rain		Not given	Not given		
		Case 5-spleen, Liver, lungs, blood		Not given	Not given		Septic shock, ARDS, death
		Case 6- mediastinum	Oral co-amoxiclav and Co-trimoxazole	Not given	Not given		Lost follow up
		Case 7- spleen, blood	Oral co-amoxiclav and Co-trimoxazole	Not given	14 weeks		Symptoms free at 6 months
Nandasiri et al. [41]	Case report	Spinal cord, bones, psoas abscess	Oral Co-trimoxazole oral doxycycline	Not given	52 weeks		The residual neurological deficits including the paraplegia, complete sensory loss and sphincter disturbance persisted
Weerasinghe et al. [42]	Case report	Bone- hip joint	Oral Co-trimoxazole	Not given	10 weeks		Symptoms resolved
Owen et al. [43]	Case report	Brain	Oral Co-trimoxazole	Not given	Not given		
Shrestha et al. [44]	Case report	Case 1—liver, soft tissues, lungs	Oral doxycycline and oral Co-trimoxazole	oral doxycycline [100 mg 12 hourly] and oral Co-trimoxazole (960 mg once daily) for three months	12 weeks		
		Case 2- spleen	Oral doxycycline and oral Co-trimoxazole		12 weeks		
Karunarathna et al. [45]	Case report	Bones, soft tissues	Oral Co-trimoxazole		24 weeks		Symptoms resolved
Martin et al. [46]	Case report	Liver	Oral Co-trimoxazole	Oral co- trimoxazole 800/160 mg twice a day	12 weeks		Symptoms resolved
Phillips et al. [47]	Case report	Sinuses	Oral Co-trimoxazole (Bactrim®)	Oral co-trimoxazole (Bactrim^®^) 160 mg/800 mg twice daily for 14 weeks	14 weeks		Culture negative for B. psuedomallei
Mabayoje et al. [48]	Case report	Knee joint	Oral doxycycline and Oral Co-trimoxazole	Oral co-trimoxazole (960 mg 2 × /d; 160 mg of trimethoprim and 800 mg of sulfamethoxazole) and doxycycline (100 mg 2 × /d)	8 weeks		Had full range of movements of the knee joints
Huang et al. [49]	Case report	Joints, lungs	Oral Co-trimoxazole	960 mg tablets of oral Co-trimoxazole every 12hourly	24 weeks		Symptoms resolved and no relapses occurred
Jayawardena et al. [50]	Case report	Soft tissues	Oral Co-trimoxazole	Oral co-trimoxazole 960 mg 12-hourly	24 weeks		Symptoms resolved
Rahim et al. [51]	Case reports	Kidney	Oral doxycycline and Oral Co-trimoxazole	Oral co-trimoxazole (960 mg Q 12 h) and doxycycline (100 mg Q 12 h)	20 weeks		Symptoms resolved and no relapses occurred
Zaw et al. [52]	Case report	Lungs	Oral Co-trimoxazole	Oral co-trimoxazole (800 mg/160 mg) 2 tablets twice a day with daily folic acid5mg	12 weeks		Symptoms resolved
Nair et al. [53]	Case report	Ankle joint	Oral Co-trimoxazole	Oral co-trimoxazole 160 mg/800 mg	20 weeks		Normal weight bearing in 6 months
Soo et al. [21]	Case report	Lungs	Oral doxycycline and Oral Co-trimoxazole	oral doxycycline 100 mg 12 hourly and three tablets of co-trimoxazole 80/400 mg (TMP-SMX) 12hourly	20 weeks		Symptoms resolved
Commons et al. [54]	Case report	Lungs	Oral Co-trimoxazole	Oral co-trimoxazole 1600/320 mg bd	6 weeks given (intended duration 3 months)	Agitation, exacerbation of psoriatic skin lesions and thrush	Due to adverse effects drug changed into amoxicillin-clavulanic acid (500/125 mg 3mane, 2 midi, 3 nocte).1The patient continued this treatment for the remaining 6 weeks of eradication therapy. Then symptoms resolved
Wijekoon et al. [55]	case report	Liver, spleen and CSF	Oral Co-trimoxazole and Co-amoxiclav	Oral co-trimoxazole 1920 mg 12hourly and co-amoxiclav 625 mg 8 h	12 weeks		No relapse or weakness
Pitman et al. [67]	Case report	Brain and bones	Oral Co-trimoxazole	Oral co-trimoxazole (TMP-SMX) at the dose of 3 × 80 mg of TMP–400 mg of SMX (480 mg), every 12 h	24 weeks		No Neurological deficits occurred and symptoms were resolved
Antony et al. [56]	Case report	Central nervous system	Oral Co-trimoxazole	Oral co-trimoxazole (TMP 320 mg/SMX1600mg) two times per day	12 weeks		The facial palsy showed improvement
Kuijpers et al. [57]	Case report	Skin and soft tissues	Oral Co-trimoxazole	oral trimethoprim–sulfamethoxazole 1920 mg every 12 h	6 weeks / 3 months		No recurrence of the skin abscess was observed
Ding et al. [58]	case report	Left infrarenal aortic aneurysm	Oral Co-trimoxazole and doxycycline	Co-trimoxazole (320 mg/1600 mg) bd and oral doxycycline 100 mg bd	20 weeks		Symptoms resolved
Sachindra et al. [59]	Case report	Lungs, kidneys and brain	Oral Co-trimoxazole	Co-trimoxazole (1920 mg 12 hourly	12 weeks		Resolved completely
Redondo et al. [60]	Case report	Bones	Oral Co-trimoxazole and doxycycline	oral doxycycline (100 mg every 12 h) and Oral co-trimoxazole (1double strength tablet every 12 h)	48 weeks		Symptoms resolved
Vaid et al. [61]	Case report	Temporomandibular joint (TMJ)	Oral Co-trimoxazole	Oral co-trimoxazole 240/1200 mg orally twice a day	24 weeks		Symptoms resolved
Saonanon,et al. [62]	Case report	Case 2-Orbit	Oral Co-trimoxazole and doxycycline	Co-trimoxazole (80/400 mg) 2 tablets 3 times a day in combination with doxycycline (100 mg) 1 tablet twice a day	24 months		The patient had showed no relapse, OD visual acuity was 20/30 and he had a noticeable scar on his forehead
Lee et al. [63]	Case report	Case 1—Liver, spleen	Oral Co-trimoxazole		32 months		No relapse for 2 years
		Case 2—Liver, spleen, prostate, lungs	Oral Co-trimoxazole	Oral Use co-trimoxazole (TMP 80 mg; SMX400 mg) two tablets every 6 h	6 weeks		Patient was clinically well
Svensson et al. [64]	Case report	Soft tissues	Oral Co-trimoxazole and doxycycline	Oral eradication therapy with doxycycline (100 mg) and Oral Co-trimoxazole (160 mg/800 mg) twice daily was then started	20 weeks	nausea	No recurrence observed
Shrestha et al. [65]	Case report	Lungs	Oral Co-trimoxazole (TMP/SMX)	Oral co-trimoxazole (TMP/SMX) 10 mg/kg	16 weeks		Symptoms resolved
Bodilsen et al. [66]	Case report	Joints	Oral Co-trimoxazole	Oral co-trimoxazole TMP-SMX 800/160 mg twice daily	12 weeks		Wound healed without a scar and no history of relapse
Behera et al. [67]	Case report	Liver, spleen, lungs and joints	Oral Co-trimoxazole (MIC < 2/38 microg/mL)	Oral co-trimoxazole (TMP/SMX) 1 double strength tablet every 12 h	24 weeks		Symptoms resolved
Saravu et al. [68]	Case report	Case 1—brain, lung, liver, spleen	Oral doxycycline and oral Co-trimoxazole	Oral co-trimoxazole 320 mg/1600 mg, twice a day and doxycycline 100 mg every 12 h	24 weeks		Patient improved
		Case 2—spinal cord	Oral doxycycline and oral Co-trimoxazole	Oral co-trimoxazole 320 mg/1600 mg, twice a day and doxycycline 100 mg every 12 h	24 weeks		Patient improved partially

## Results

**Fig. 1:**
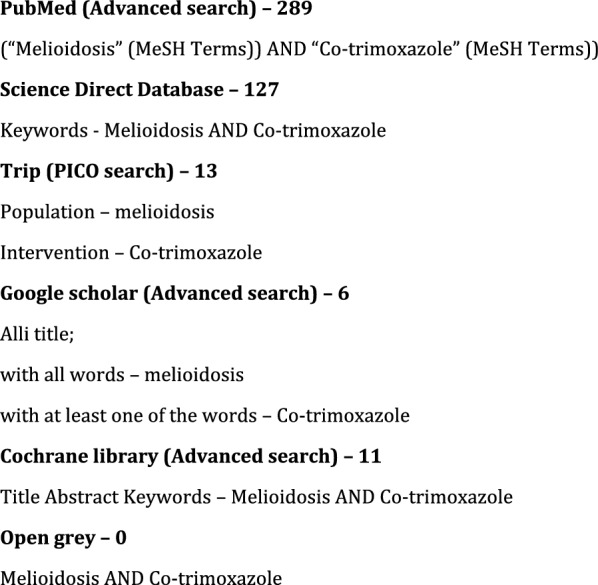
The search strategies and the number of results in each database

**Fig. 2:**
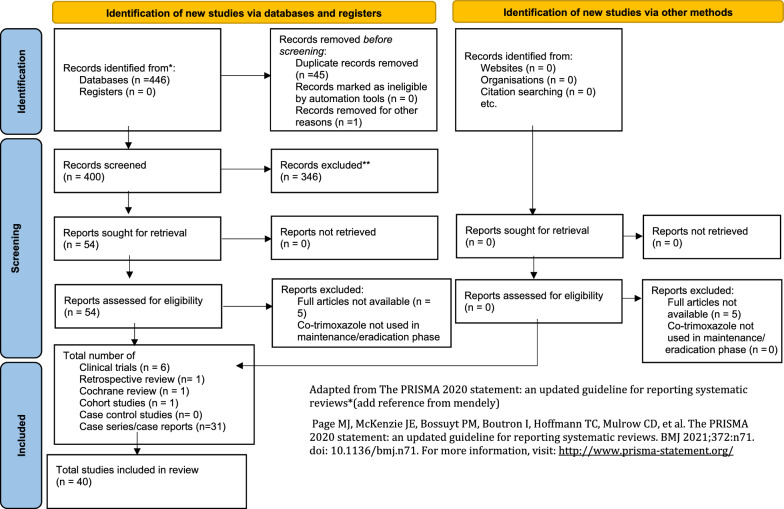
PRISMA flow diagram of the review with number of results at each step

A total of 40 results were found from the searched databases, and no article was selected from their reference list (Fig. 1). The PRISMA search returned four hundred and forty-six (446) articles, forty-six (46) of which were removed as duplicates. After removing duplicates, 400 articles were included for the title and abstract screening. Fifty-four articles were found eligible for full article screening. Figure 2 PRISMA flow diagram presents the number of articles in each step [33].

### The focus of infection and antimicrobial therapy

Melioidosis commonly affects the respiratory system leading to pneumonia, pleural effusion, and lung abscesses. In the eradication phase, co-trimoxazole was used following respiratory melioidosis. Mostly the patients were treated with 960 mg of oral co-trimoxazole every 12 h for 3–6 months (Additional file 1: Table S1), and patients clinically improved after treatments. In gastrointestinal melioidosis the liver and spleen were the most affected organs, and, in those patients, co-trimoxazole was given alone or as a combination with doxycycline in the eradication phase. These patients were treated for up to 6 months, and most recovered without relapsing (Additional file 1: Table S1).

In melioidosis develops in brain, co-trimoxazole was given for six months to one year, and after the therapy, no residual neurologic deficits were detected, and patients recovered completely (Additional file 1: Table S1). When *Burkholderia pseudomallei* invades the spinal cord of patients causing transverse myelitis, patients were given co-trimoxazole and doxycycline combination in the eradication phase. Oral co-trimoxazole 320 mg/1600 mg, twice daily, and doxycycline 100 mg every 12 h, given for six months to one year [17, 19]. After one year of therapy patient with transverse myelitis had residual neurological deficits, including paraplegia, complete sensory loss, and sphincter disturbance [17].

When melioidosis is developed in the bones and joints, they commonly presented with septic arthritis and osteomyelitis. They were also treated with oral co-trimoxazole alone and in combination with doxycycline for more than ten weeks in the eradication phase. Patients fully recovered with a full range of movements and without relapses (Additional file 1: Table S1).

There were case reports of orbital cellulitis and necrotizing fasciitis following melioidosis which was treated with oral co-trimoxazole (960 mg) three times a day in combination with doxycycline100mg twice a day for six months. The patient had shown no relapses but had a slight reduction of visual acuity [18]. In genitourinary melioidosis, oral co-trimoxazole was given for less than 20 weeks in the eradication phase [23, 24].

**Fig. 3:**
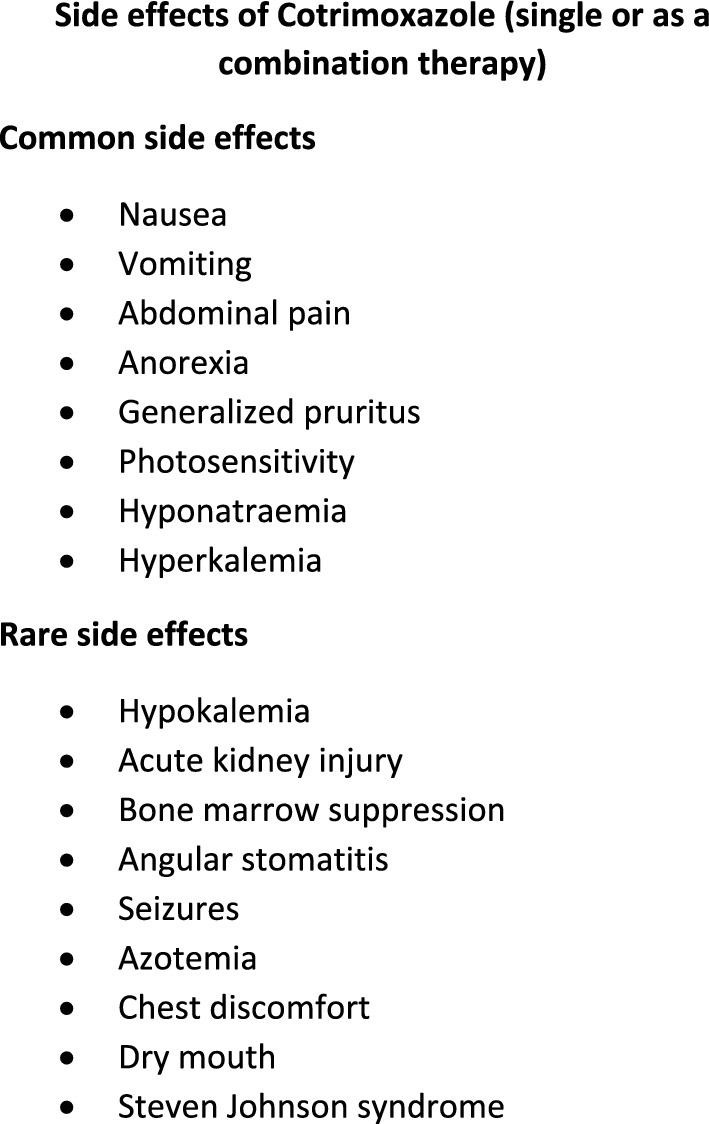
Adverse effects of co-trimoxazole

When reporting the adverse effects agitation, exacerbation of psoriatic skin lesions, and thrush [16] were observed in patients who used co-trimoxazole only therapy in the eradication phase, and weight gain was [29] observed in patients who used oral amoxicillin-clavulanate and co-trimoxazole combination. The list of adverse effects is given in Fig. 3.

### Antimicrobial therapy (monotherapy vs combinations)

**Fig. 4:**
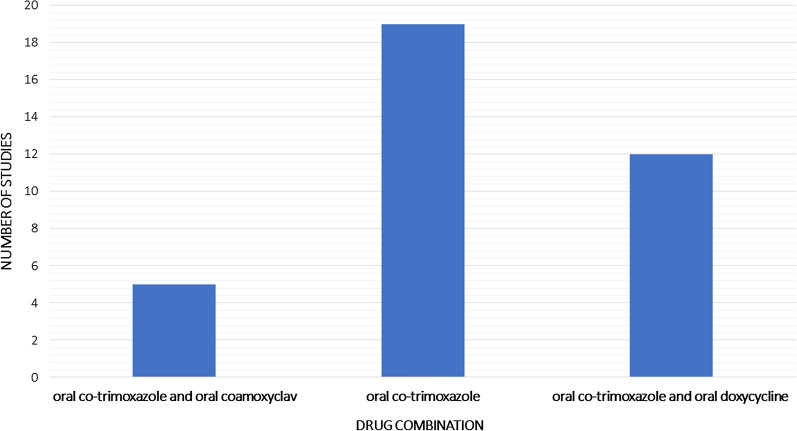
Different drug combinations used in case series and reports

There were 6 controlled trials, 1 cohort, and 1 Cochrane review where Co-trimoxazole was used in the maintenance phase. In one RCT co-trimoxazole was used alone; in others (n = 5), it was used in combination with doxycycline (Table 2). In Chetchotisakd et al. [37] co-trimoxazole monotherapy (co-trimoxazole with placebo) was compared with co-trimoxazole combination therapy. Although co-trimoxazole was given via the oral route in all studies, there were variations in the duration of antibiotic therapy among studies (Fig. 4).

**Table 2:** Summary of the RCTs using co-trimoxazole in the eradication phase

Name	Study type	Drugs	Sample size	Open or blind	Dose	Duration of eradication phase	Relapse rate		Mortality rate
							Culture positive	Clinical relapse	
Chaowagul et al. [34]	RCT	1.Co-trimoxazole, doxycycline, chloramphenicol	91	Open	Co- trimoxazole (8mg TMP and 40mg SMX/kg/ daily; max 160mg TMP and 800mg SMX/twice daily	12 weeks	11.8%		14%
		2.Co-trimoxazole (TMP/SMX), doxycycline	89				5.4%		11%
Rajchanuvong et al. [35]		1.Chloramphenicol, doxycycline, Co-trimoxazole	52	Open	Co-trimoxazole (10mg TMP + 50mg SMX/kg/day in 2 divided doses)	20 weeks	10%		1.92%
		2. Doxycycline	49				36%		8.16%
Anunnatsiri et al. [36]	RCT	Co-trimoxazole	12 week – 322	Open	< 40 kg, the dose used was 160/800 mg of TMP/SMX twice daily; for a body weight of 40–60 kg, the dose was 240/1200 mg of TMP/SMX twice daily; and for a body weight > 60 kg, the dose was 320/1600 mg of TMP/SMX twice daily	12 weeks	2%		0.3%
			20 weeks			20 weeks	1%		3%
Chetchotisakd et al. [37]	RCT	1.Co-trimoxazole, placebo	311	Double blind	TMP-SMX (80 mg TMP and 400 mg SMX) tablets were prescribed using a weight-based dose, bodyweight less than 40 kg or eGFR 15–29 mL/min, 160 mg TMP and 800 mg SMX twice daily; bodyweight of 40 kg to 60 kg, 240 mg TMP and 1200 mg SMX twice daily; and bodyweight greater than 60 kg, 320 mg TMP and 1600 mg SMX twice daily	20 weeks	16%	3%	3%
		2.Co-trimoxazole, Doxycycline	315				21%	8%	1%
Chetchotisakd et al. [4]	RCT	1. Ciprofloxacin and azithromycin	32	Open	Co- trimoxazole (10mg TMP + 50mg SMX/kg/day in 2 divided doses)	12 weeks	22%		3.12%
		2. Co-trimoxazole and doxycycline	33			20 weeks	3%		0%
Chaowagal et al. [38]	RCT	1. Co-trimoxazole, doxycycline, chloramphenicol	91	Open	Co- trimoxazole (8mg TMP and 40mg SMX/kg/ daily; max 160mg TMP and 800mg SMX/twice daily	12 weeks	2%	5.9%	4.4%
		2.Doxycycline	89				5.6%	13%	6.74%
Chaowagul et al. [34]	RCT	1.Co-trimoxazole, doxycycline, chloramphenicol	91	Open	Co- trimoxazole (8mg TMP and 40mg SMX/kg/ daily; max 160mg TMP and 800mg SMX/twice daily	12 weeks	11.8%		14%
		2.Co-trimoxazole (TMP/SMX), doxycycline	89				5.4%		11%
Rajchanuvong et al.[35]		1.Chloramphenicol, doxycycline, Co-trimoxazole	52	Open	Co-trimoxazole (10mg TMP + 50mg SMX/kg/day in 2 divided doses)	20 weeks	10%		1.92%
		2. Doxycycline	49				36%		8.16%
Anunnatsiri et al. [36]	RCT	Co-trimoxazole	12 week – 322	Open	< 40 kg, the dose used was 160/800 mg of TMP/SMX twice daily; for a body weight of 40–60 kg, the dose was 240/1200 mg of TMP/SMX twice daily; and for a body weight > 60 kg, the dose was 320/1600 mg of TMP/SMX twice daily	12 weeks	2%		0.3%
			20 weeks			20 weeks	1%		3%
Chetchotisakd et al. [37]	RCT	1.Co-trimoxazole, placebo	311	Double blind	TMP-SMX (80 mg TMP and 400 mg SMX) tablets were prescribed using a weight-based dose, bodyweight less than 40 kg or eGFR 15–29 mL/min, 160 mg TMP and 800 mg SMX twice daily; bodyweight of 40 kg to 60 kg, 240 mg TMP and 1200 mg SMX twice daily; and bodyweight greater than 60 kg, 320 mg TMP and 1600 mg SMX twice daily	20 weeks	16%	3%	3%
		2.Co-trimoxazole, Doxycycline	315				21%	8%	1%
Chetchotisakd et al. [4]	RCT	1. Ciprofloxacin and azithromycin	32	Open	Co- trimoxazole (10mg TMP + 50mg SMX/kg/day in 2 divided doses)	12 weeks	22%		3.12%
		2. Co-trimoxazole and doxycycline	33			20 weeks	3%		0%
Chaowagal et al. [38]	RCT	1. Co-trimoxazole, doxycycline, chloramphenicol	91	Open	Co- trimoxazole (8mg TMP and 40mg SMX/kg/ daily; max 160mg TMP and 800mg SMX/twice daily	12 weeks	2%	5.9%	4.4%
		2.Doxycycline	89				5.6%	13%	6.74%

Another RCT [36] compared different durations of co-trimoxazole single therapy during the eradication phase of melioidosis. All other five controlled trials compared two sets of drugs, at least one set containing co-trimoxazole (Table 2). Eight different co-trimoxazole-containing drug combinations have been used in these six trials (Table 2 gives the eight combinations).

Three studies used co-trimoxazole, doxycycline, and chloramphenicol [7–9]; however, the comparison varied significantly (Table 2). Three studies used co-trimoxazole and doxycycline [7, 10, 11], and one study used co-trimoxazole with a placebo [10]. Some studies compared the treatment with drug combinations without having co-trimoxazole. One study used co-amoxiclav [8], two studies used doxycycline [9, 12], and one study used ciprofloxacin and azithromycin [11] in their eradication phase.

Of the case reports, 37/41 co-trimoxazole used in their eradication phase. There were 19 cases in which used co-trimoxazole alone while in 12 cases oral co-trimoxazole and doxycycline combination was used. There were 5 cases with oral co-trimoxazole and amoxicillin clavulanate combination (Additional file 1: Table S1).

There was a patient with a loss of follow-up [29] and one patient changed the antimicrobial (co-trimoxazole to co-amoxiclav) due to adverse effects (Table 3) [16].

**Table 3:** Different drug regimens and the numbers of studies/case reports with each drug regimens

Dose and regimen as given in the article	Randomized control trials	Case reports and case series	Cohort study
Oral co-trimoxazole 960 mg (Trimethoprim/Sulfamethoxazole) once daily	0	04	0
Oral co-trimoxazole 960 mg (Trimethoprim/Sulfamethoxazole) twice daily	02	10	0
Oral co-trimoxazole 1920 mg (Trimethoprim/Sulfamethoxazole) twice daily	02	10	1
Oral co- trimoxazole (10mg TMP + 50mg SMX/kg/day in 2 divided doses)	02	0	0
Oral co-trimoxazole 240/1200 mg (Trimethoprim/Sulfamethoxazole) orally twice a day	0	02	0
Oral co-trimoxazole 10 mg/kg (Trimethoprim/Sulfamethoxazole)	0	01	0

### Duration of eradication phase and dosage of antimicrobials

Duration of treatment also varied for different combinations of co-trimoxazole in which 50 percent (four out of eight) had 12 weeks of treatments [7, 9, 11, 13] while the remaining had 20 weeks of treatments [8, 11, 13].

Co-trimoxazole dose in the maintenance phase varies in 6 studies. There were three variations. Two studies used co-trimoxazole 960 mg (160 mg trimethoprim and 800 mg sulfamethoxazole) twice daily regimen [7, 9]. Two studies used co-trimoxazole 1920 mg twice-daily regimen [10, 13], and two studies used co-trimoxazole 60 mg/kg/day in two divided doses [8, 11].

**Fig. 5:**
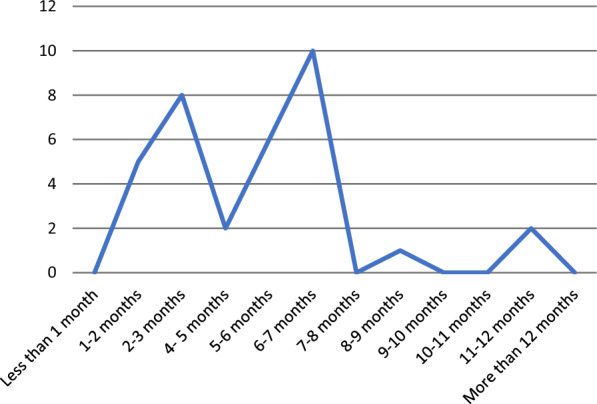
Duration of eradication phase in different studies

The primary outcomes of all RCTs were to assess the mortality and relapse rates. Relapses were due to microbial failure and treatment failure. When using co-trimoxazole alone, culture positive relapse rate was 2% [13] and 1.16% [10]. The clinical relapse rate was 3% in Chetchotisakd et al*.* [4, 10]. When using co-trimoxazole as a combination therapy culture positive relapse rate vary in between 2 and 21% (Table 2), and the clinical relapse rate varies between 2 and 15.9% (Table 2). It also had 9% of treatment failure [11]. Other drugs and combinations showed culture positive relapse rate of 22–36% (Table 2), treatment failure of 28% [11], and clinical relapse of 13% [9] (Fig. 5).

Mortality in patients with melioidosis after eradication therapy varies between studies. In co-trimoxazole alone, therapy mortality differs from 0.3 to 3% (Table 2) while co-trimoxazole combination therapy is 0–14% (Table 2) and other drug combinations 3.12–8.16% (Table 2).

When considering the duration of the eradication phase, varies from 2 months [20] to one year [21, 22, 29]. In most cases, patients were given antimicrobials for six months. None of the reported cases has been given co-trimoxazole for more than one year or less than one month in the eradication phase (Additional file 1: Table S1).

### Outcome following therapy

There were two observational studies, [28] was a retrospective review, and [27] is a retrospective cohort study. In [28], co-trimoxazole, doxycycline combination, and co-trimoxazole alone were reviewed. Oral co-trimoxazole 960 mg (160 mg trimethoprim and 800 mg sulfamethoxazole) was given twice daily for 20 weeks. In combination therapy culture positive relapse rate was 4.6%, and the clinical recurrence rate was 2%, with a mortality rate of 0.9%. On co-trimoxazole alone, culture positive relapse rate was 3.2%, and the clinical recurrence rate was 1% [14].

In Ref. [27]*,* 212 patients were selected for the cohort study, and from them, 95.8% were commenced on co-trimoxazole monotherapy, 2.8% were on doxycycline and 1.4% had no oral therapy. Of those, 88.7% received treatments for 3 months and 9.9% received 6 months or greater. Oral co-trimoxazole 1920 mg was used twice daily. Following co-trimoxazole therapy, there were 2.8% recrudescence and 4.2% recurrences [12].

In 28 cases, patients were cured without any residual abnormalities. There were 3 case reports reported no residual abnormalities following treatment cessation and from that 2 patients were diagnosed with melioidosis in the central nervous system [17–19]. There were no identified relapses in any case report, even though 2 patients were followed up for two years [20, 22]. There were no reported deaths due to melioidosis in patients who were treated with co-trimoxazole in the eradication phase.

### Adverse effects of antimicrobial therapy

With regard to adverse effects of co-trimoxazole alone, anemia, hyponatremia, hyperkalemia, rarely hypokalaemia, severe hyponatremia, gastrointestinal side effects [8, 13], acute kidney injury, bone marrow suppression and rash were reported and [12] some patients changed the antimicrobial or opted to reduce the dose. Use of co-trimoxazole combination therapy reported nausea, vomiting or abdominal pain [7, 9–11], rash [7–10], photosensitivity [7–9], anemia [7, 11], angular stomatitis [7], anorexia, chest discomfort, dry mouth, seizures, azotemia [7], generalized pruritus [8], Steven Johnson syndrome, severe hyponatremia, severe hyperkalemia [11] and facial erythema [9] which also led to antibiotic dose reduction, change of antibiotic and loss of follow up.

### Quality assessment

Six randomized controlled trials were assessed using the Cochrane Risk of Bias assessment tool (Additional file 2: Table S2). All of the studies introduced at least one form of bias, but the overall risk was low in 4 studies. One study had a low risk of bias in 04 criteria and one criterion with some concerns [37]. Of the remaining studies, three studies have an overall low risk of bias [4, 34, 36] and two studies have some concerns [34, 38].

To assess the cohort and cross-sectional studies, the NIH quality tool was used (Table 4) [27, 28]. According to the raters, both of the studies were fair in terms of risk of bias.

**Table 4:** Summary of Scores for NIH Quality Assessment Tool for Observational Cohort and Cross-Sectional Studies

No	Criteria	First authors of the selected articles	
		[27]	[28]
1	Was the research question or objective in this paper clearly stated?	Y	N
2	Was the study population clearly specified and defined?	Y	Y
3	Was the participation rate of eligible persons at least 50%?	NA	NA
4	Were all the subjects selected or recruited from the same or similar populations (including the same time period)? Were inclusion and exclusion criteria for being in the study prespecified and applied uniformly to all participants?	Y	NA
5	Was a sample size justification, power description, or variance and effect estimates provided?	N	N
6	For the analyses in this paper, were the exposure(s) of interest measured prior to the outcome(s) being measured?	NA	NA
7	Was the timeframe sufficient so that one could reasonably expect to see an association between exposure and outcome if it existed?	NA	NA
8	For exposures that can vary in amount or level, did the study examine different levels of the exposure as related to the outcome (e.g., categories of exposure, or exposure measured as continuous variable)?	N	N
9	Were the exposure measures (independent variables) clearly defined, valid, reliable, and implemented consistently across all study participants?	NA	Y
10	Was the exposure(s) assessed more than once over time?	N	N
11	Were the outcome measures (dependent variables) clearly defined, valid, reliable, and implemented consistently across all study participants?	Y	CD
12	Were the outcome assessors blinded to the exposure status of participants?	NA	NA
13	Was loss to follow-up after baseline 20% or less?	NA	NA
14	Were key potential confounding variables measured and adjusted statistically for their impact on the relationship between exposure(s) and outcome(s)?	CD	CD
	Overall Risk of Bias	Some concerns	Some concerns

*Y* Yes, *N* No, *CD* Cannot Determine, *NA* Not Applicable, *NR* Not Reported

Thirty-one studies were assessed using the NIH quality assessment tool for the case series (Additional file 2: Table S2). According to the two raters, seventeen studies were good, and all the other studies were fair studies when considering the risk of bias.

## Discussion

A phase of aggressive intravenous therapy and a phase of oral eradication is used to treat melioidosis. For many years co-trimoxazole has been considered one of the main drugs used in both phases. Some research articles specify the durations and doses for intravenous therapy [67]. However, they have reported poor adherence to eradication therapy due to adverse effects and in some melioidosis endemic countries have decentralized healthcare facility leading to long distance travel for the follow up. And suggested further research evaluating the duration and necessity of drug regimens of the eradication phase for different forms of melioidosis [67, 68]. Current recommendations propose commencing the intensive phase of treatment with 10 to 14 days of intravenous antibiotics for melioidosis without a focus of infection while 1–28 days or even more with a focus of infection and continuing it with 3 to 6 months of oral antibiotics (eradication phase) [69]. However, these recommendations are not based on the results of recent systematic reviews on eradication therapy [14, 70].

We found that in RCTs, co-trimoxazole monotherapy or in combinations has been tested against co-trimoxazole-containing combinations and combinations without co-trimoxazole. The case reports also have reported different combinations of co-trimoxazole with other drugs. Out of all combinations, oral co-trimoxazole and doxycycline combination is the most frequently used combination in eradication therapy.

Both mortality and relapse rate of melioidosis is higher when using co-trimoxazole as a combination therapy compared to as a monotherapy in the eradication phase [36] (Table 2). The reasons for high mortality rate following combination therapy would be an outcome of high relapses, drug toxicity, high cost and confusion of taking proper medication leading to poor compliance. We note that poor adherence and dropouts to follow the given drug regimen are comparatively higher when the number of drugs in combination is high [34].

However, the number of studies using co-trimoxazole as monotherapy was found only in two clinical trials, two observational studies, and 19 cases. Therefore, the authors believe conclusions based on these may be due to lack of evidence. On the other hand, we could not get an idea about relapse rates by studying case series and case reports. In the case of reports, there is usually no follow-up, so there may be underreporting. This may lead to publication bias. However, those are useful to ascertain adverse effects following antimicrobials.

Altogether six co-trimoxazole dosages were used in studies (Table 2) trimethoprim: Sulfamethoxazole 1:5 (320/1600 mg) combination is the frequently used dose. The 1:5 (320/1600 mg) ratio showed less mortality and low relapses when compared to the 1:5 (160/800 mg) [4] and 1:5 (10/50 mg/kg/day) [38] regimens. This will be an eye opener for the clinicians to re-think about the proper dose to achieve a cure without complications.

The duration of the eradication phase ranged from 2 months to one year, of these, co-trimoxazole 1920 mg twice daily for 3 months showed lesser mortality (0.3- 3%), microbial relapse rates (1–2%), and co-trimoxazole 960 mg twice daily dose showed less clinical relapse (1–3%) (Table 2). The current guidelines advise about the treatment duration as 3 months without a focus of infection or even one year with meningitis, brain abscess, bone and joint infections and spinal infection. The poor outcome was associated following short duration of therapy would be due to bacterial sequestration in multiple foci and host immune status [39]. Interestingly the case series and case reports have reported a much longer duration of treatment with co-trimoxazole. Redondo et al. [11] reported 12 months of treatment with the twice-daily regimen, which eventually accounts for more than a 2000 kg cumulative dose for bone infections with melioidosis. The Darwin guidelines recommend only a six-month eradication phase for bone infection. Substantial rates of adverse effects to oral co-trimoxazole seen in this study most likely reflect this high dose used for melioidosis. The adverse event profiles were, in most cases, only able to obtain qualitative data from RCTs. Therefore, a quantitative data synthesis of the occurrence of adverse events was unsuccessful to achieve in this review.

The mortality rate and relapse rate also differ according to the co-trimoxazole dose. The highest culture-positive relapse rate occurs when using co-trimoxazole in 60 mg /kg/day in 2 divided doses than the other two combinations. The lowest relapse rate and lowest mortality rate occur when using co-trimoxazole 1920 mg twice daily. The highest mortality rate was recorded when using oral co-trimoxazole 960 mg twice daily (Table 2).

The dearth of RCTs and case reports is one of the most prominently mentioned weaknesses in the papers considered for this evaluation. Selection bias, recall bias, inadequate confounding control, and exposure misclassification are further drawbacks. The eradication phase, multiple dropouts, and failure to follow a standard protocol for the treatment of melioidosis were all significant faults in the trials. Because there were so many different study designs and methodologies, it was difficult to do a quantitative analysis of the results.

## Conclusions

The dose of co-trimoxazole, duration of the eradication phase, and other combinations used in the treatment was varying between studies. Compared to combined therapy patients treated with co-trimoxazole alone the mortality and relapse rates were low. The lowest relapse rate and lowest mortality rate occur when using co-trimoxazole 1920 mg twice daily. The duration of therapy varies on the focus of melioidosis and it is ranged from 2 months to one year and minimum treatment duration associated with low relapse rate is 3 months. The use of co-trimoxazole over the maintenance phase of melioidosis is associated with clinical cure but has adverse effects.

### Supplementary Information

**Additional file 1: Table S1.** Summary of case reports where co-trimoxazole is used as eradication therapy.

**Additional file 2: Table S2.** NIH quality assessment tool for the case series studies.

## Data Availability

All data generated or analyzed during the study are included in this published article (and its additional files).

## Abbreviations

RCT: Randomised controlled trial
SMX: Sulfamethoxazole
TMP: Trimethoprim
HR: Hazardous ratio
PRISMA: Preferred reporting items for systematic reviews and meta-analyses
