# Supplementary material for: Efficacy and safety of co-trimoxazole in eradication phase of melioidosis; systematic review
Source: Ann Clin Microbiol Antimicrob. 2023 Aug 17;22:74. doi: 10.1186/s12941-023-00620-z (PMC10436656; doi:10.1186/s12941-023-00620-z)
Supplement: Supplementary file 1 — Additional file 1: Table S1. Summary of case reports where co-trimoxazole is used as eradication therapy. [file 12941_2023_620_MOESM1_ESM.docx]

**Table S1. Summary of case reports where co-trimoxazole is used as eradication therapy**

| **Study** | **Study**  **design** | **Site**  **of**  **infection** | **Drugs used**  **in**  **eradication phase** | **Dose** | **Duration** | **Side effects** | **Outcomes** |
| --- | --- | --- | --- | --- | --- | --- | --- |
| Saravu *et. al*. (2008)(29) | Case series | Case 1 - liver, blood, lungs |  | Not given | Not given |  |  |
|  |  | Case 2- liver, spleen, lungs, bones | Oral co-amoxiclav and Oral Co-trimoxazole | Not given | 24 weeks | Weight gain | Splenomegaly completely regressed; hip pain subsided |
|  |  | Case 3-liver, spleen |  | Not given | Not given |  |  |
|  |  | Case 4- liver, spleen, rain |  | Not given | Not given |  |  |
|  |  | Case 5-spleen, Liver, lungs, blood |  | Not given | Not given |  | Septic shock, ARDS, death |
|  |  | Case 6- mediastinum | Oral co-amoxiclav and Co-trimoxazole | Not given | Not given |  | Lost follow up |
|  |  | Case 7- spleen, blood | Oral co-amoxiclav and Co-trimoxazole | Not given | 14 weeks |  | Symptoms free at 6 months |
| Nandasiri et al (2012)(39) | Case report | Spinal cord, bones, psoas abscess | Oral Co-trimoxazole oral doxycycline | Not given | 52 weeks |  | The residual neurological deficits including the paraplegia, complete sensory loss and sphincter disturbance persisted. |
| Weerasinghe et al (2018)(40) | Case report | bone- hip joint | Oral Co-trimoxazole | Not given | 10 weeks |  | Symptoms resolved |
| Owen et al (2020)(41) | Case report | Brain | Oral Co-trimoxazole | Not given | Not given |  |  |
| Shrestha et al (2019)(42) | Case report | Case 1 - liver, soft tissues, lungs | Oral doxycycline and oral Co-trimoxazole | oral doxycycline (100 mg 12 hourly) and oral Co-trimoxazole (960 mg once daily) for three months | 12 weeks |  |  |
|  |  | Case 2- spleen | Oral doxycycline and oral Co-trimoxazole |  | 12 weeks |  |  |
| Karunarathna et al (2018)(43) | Case report | Bones, soft tissues | Oral Co-trimoxazole |  | 24 weeks |  | Symptoms resolved |
| Martin et al (2016)(44) | Case report | Liver | Oral Co-trimoxazole | Oral co- trimoxazole 800/160 mg twice a day | 12 weeks |  | Symptoms resolved |
| Phillips et al (2016)(45) | Case report | Sinuses | Oral Co-trimoxazole (Bactrim®) | Oral co-trimoxazole (Bactrim®) 160 mg/800 mg twice daily for 14 weeks | 14 weeks |  | Culture negative for B. psuedomallei |
| Mabayoje et al (2022)(46) | Case report | Knee joint | Oral doxycycline and Oral Co-trimoxazole | Oral co-trimoxazole (960 mg 2×/d; 160 mg of trimethoprim and 800 mg of sulfamethoxazole) and doxycycline (100 mg 2×/d) | 8 weeks |  | Had full range of movements of the knee joints |
| Huang et al (2018)(47) | Case report | Joints, lungs | Oral Co-trimoxazole | 960mg tablets of oral Co-trimoxazole every 12hourly | 24 weeks |  | Symptoms resolved and no relapses occurred |
| Jayawardena et al (2019)(48) | Case report | Soft tissues | Oral Co-trimoxazole | Oral co-trimoxazole 960 mg 12-hourly | 24 weeks |  | Symptoms resolved |
| Rahim et al (2018)(49) | Case reports | Kidney | Oral doxycycline and Oral Co-trimoxazole | Oral co-trimoxazole (960 mg Q 12h) and doxycycline (100 mg Q 12h) | 20 weeks |  | Symptoms resolved and no relapses occurred |
| Zaw et al (2019)(50) | Case report | Lungs | Oral Co-trimoxazole | Oral co-trimoxazole (800mg/160mg) 2 tablets twice a day with daily folic acid5mg | 12 weeks |  | symptoms resolved |
| Nair et al (2017)(51) | Case report | Ankle joint | Oral Co-trimoxazole | Oral co-trimoxazole 160 mg/800 mg | 20 weeks |  | Normal weight bearing in 6 months |
| Soo et al (2015)(17) | Case report | Lungs | Oral doxycycline and Oral Co-trimoxazole | oral doxycycline 100 mg 12 hourly and three tablets of co-trimoxazole 80/400 mg (TMP-SMX) 12hourly | 20 weeks |  | symptoms resolved |
| Commons et al (2014)(52) | Case report | Lungs | Oral Co-trimoxazole | Oral co-trimoxazole 1600/320 mg bd | 6 weeks given. ( intended duration 3 months) | Agitation, exacerbation of psoriatic skin lesions and thrush | Due to adverse effects drug changed into amoxicillin-clavulanic acid (500/125 mg 3mane, 2 midi, 3 nocte).1The patient continued this treatment for the remaining 6 weeks of eradication therapy. Then symptoms resolved. |
| Wijekoon et al (2016)(53) | case report | Liver, spleen and CSF | Oral Co-trimoxazole and Co-amoxiclav | Oral co-trimoxazole 1920 mg 12hourly and co-amoxiclav 625 mg 8 hour | 12 weeks |  | No relapse or weakness |
| Amarendra et al (2021) | Case report | Brain and bones | Oral Co-trimoxazole | Oral co-trimoxazole (TMP-SMX) at the dose of 3×80 mg of TMP–400 mg of SMX (480 mg), every 12 hour | 24 weeks |  | No Neurological deficits occurred and symptoms were resolved |
| Antony et al (2017)(54) | Case report | Central nervous system | Oral Co-trimoxazole | Oral co-trimoxazole (TMP 320mg/SMX1600mg) two times per day | 12 weeks |  | The facial palsy showed improvement |
| Kuijpers et al (2021)(55) | Case report | skin and soft tissues | Oral Co-trimoxazole | oral trimethoprim–sulfamethoxazole 1920 mg every 12 h | 6 weeks / 3 months |  | No recurrence of the skin abscess was observed |
| Ding et al (2013)(56) | case report | left infrarenal aortic aneurysm | Oral Co-trimoxazole and doxycycline | Co-trimoxazole (320 mg/1600 mg) bd and oral doxycycline 100 mg bd | 20 weeks |  | Symptoms resolved |
| Sachindra et al (2017)(57) | Case report | Lungs, kidneys and brain | Oral Co-trimoxazole | Co-trimoxazole (1920 mg 12 hourly | 12 weeks |  | Resolved completely |
| Redondo et al (2011)(58) | Case report | Bones | Oral Co-trimoxazole and doxycycline | oral doxycycline (100 mg every 12 h) and Oral co-trimoxazole (1double strength tablet every 12 h) | 48 weeks |  | Symptoms resolved |
| Vaid et al (2015)(59) | Case report | Temporomandibular joint (TMJ) | Oral Co-trimoxazole | Oral co-trimoxazole 240/1200 mg orally twice a day | 24 weeks |  | Symptoms resolved |
| Saonanon,et al (2013)(60) | Case report | Case 2-Orbit | Oral Co-trimoxazole and doxycycline | Co-trimoxazole (80/400mg) 2 tablets 3 times a day in combination with doxycycline (100mg) 1 tablet twice a day | 24 months |  | The patient had showed no relapse, OD visual acuity was 20/30 and he had a noticeable scar on his forehead |
| Lee et al (2006)(61) | Case report | Case 1 - Liver, spleen | Oral Co-trimoxazole |  | 32 months |  | No relapse for 2 years |
|  |  | Case 2 - Liver, spleen, prostate, lungs | Oral Co-trimoxazole | Oral Use co-trimoxazole (TMP 80 mg; SMX400 mg) two tablets every 6 hours | 6 weeks |  | Patient was clinically well. |
| SVENSSON et al (2006)(62) | Case report | Soft tissues | Oral Co-trimoxazole and doxycycline | Oral eradication therapy with doxycycline (100 mg) and Oral Co-trimoxazole (160 mg/800mg) twice daily was then started. | 20 weeks | nausea | No recurrence observed |
| SHRESTHA et al (2005)(63) | Case report | Lungs | Oral Co-trimoxazole (TMP/SMX) | Oral co-trimoxazole (TMP/SMX) 10 mg/kg | 16 weeks |  | Symptoms resolved |
| Bodilsen et al (2014)(64) | Case report | Joints | Oral Co-trimoxazole | Oral co-trimoxazole TMP-SMX 800/160 mg twice daily | 12 weeks |  | Wound healed without a scar and no history of relapse |
| Behera et al (2012)(65) | Case report | Liver, spleen, lungs and joints | Oral Co-trimoxazole (MIC<2/38 microg/mL) | Oral co-trimoxazole (TMP/SMX) 1 double strength tablet every 12 h | 24 weeks |  | Symptoms resolved |
| Saravu et al (2015)(66) | Case report | Case 1- brain, lung, liver, spleen | Oral doxycycline and oral Co-trimoxazole | Oral co-trimoxazole 320 mg/1600 mg, twice a day and doxycycline 100 mg every 12 hours | 24 weeks |  | Patient improved |
