# Supplementary material for: Efficacy and safety of co-trimoxazole in eradication phase of melioidosis; systematic review
Source: Ann Clin Microbiol Antimicrob. 2023 Aug 17;22:74. doi: 10.1186/s12941-023-00620-z (PMC10436656; doi:10.1186/s12941-023-00620-z)
Supplement: Supplementary file 2 — Additional file 2: Table S2. NIH quality assessment tool for the case series studies. [file 12941_2023_620_MOESM2_ESM.docx]

**Table S2: NIH quality assessment tool for the case series studies**

|  | Sarav et. al. | Nandasiri et.al.2012 | Weerasinghe et.al. | Owen et.al. | Sherstha et.al. | Karunarathna et.al. | Martin et.al. | Phillips et.al. | Mabayoje et.al. | Huang et.al. | Jayawardena et.al. | Rahim et.al. | Zaw et.al. | Amarendra et.al. | Soo et.al. | Raja et.al. |
| --- | --- | --- | --- | --- | --- | --- | --- | --- | --- | --- | --- | --- | --- | --- | --- | --- |
| 1. Was the study question or objective clearly stated? | Y | Y | Y | Y | Y | Y | T | Y | Y | Y | Y | Y | Y | Y | Y | Y |
| 2. Was the study population clearly and fully described, including a case definition? | Y | Y | Y | Y | N | Y | Y | Y | Y | Y | Y | Y | Y | Y | Y | Y |
| 3. Were the cases consecutive? | Y | NA | NA | NA | CD | NA | NA | NA | NA | NA | NA | NA | NA | NA | NA | NA |
| 4. Were the subjects comparable? | N | NA | NA | NA | N | NA | NA | NA | NA | NA | NA | NA | NA | NA | NA | NA |
| 5. Was the intervention clearly described? | Y | Y | Y | Y | Y | Y | Y | Y | Y | Y | Y | Y | Y | CD | Y | Y |
| 6. Were the outcome measures clearly defined, valid, reliable, and implemented consistently across all study participants? | N | NA | NA | NA | CD | NA | NA | NA | NA | NA | NA | NA | NA | NA | NA | NA |
| 7. Was the length of follow-up adequate? | CD | Y | CD | Y | CD | Y | N | N | Y | Y | N | N | N | Y | Y | Y |
| 8. Were the statistical methods well-described? | NR | NR | CD | Y | N | CD | CD | CD | CD | Y | Y | CD | Y | Y | Y | CD |
| 9. Were the results well-described? | y | Y | y | Y | Y | Y | Y | Y | Y | Y | Y | Y | Y | CD | Y | Y |
| Quality rating | Fair | Good | Fair | Good | Fair | Fair | Fair | Fair | Fair | Good | Good | Fair | Good | Fair | Good | Fair |

*CD, cannot determine; NA, not applicable; NR, not reported

|  | Commons et.al. | Wijekoon et.al. | Antony et.al. | Kuijpers et.al. | Ding et.al. | Wijewickrma et.al. | Redondo et. al. | Vaid et. al. | Saonanon et.al. | Lee et. al | Svensson et.al. | Nabin et.al. | Bodilsen et.al. | Behera et.al. | Sarav et.al. |
| --- | --- | --- | --- | --- | --- | --- | --- | --- | --- | --- | --- | --- | --- | --- | --- |
| 1. Was the study question or objective clearly stated? | Y | Y | Y | Y | Y | Y | Y | Y | Y | Y | Y | Y | Y | Y | Y |
| 2. Was the study population clearly and fully described, including a case definition? | N | Y | Y | N | Y | Y | Y | Y | Y | Y | N | Y | Y | Y | Y |
| 3. Were the cases consecutive? | NA | NA | NA | NA | NA | NA | NA | NA | NA | N | NA | NA | NA | NA | N |
| 4. Were the subjects comparable? | NA | NA | NA | NA | NA | NA | NA | NA | NA | N | NA | NA | NA | NA | N |
| 5. Was the intervention clearly described? | Y | Y | Y | Y | Y | Y | Y | Y | Y | Y | Y | Y | Y | Y | Y |
| 6. Were the outcome measures clearly defined, valid, reliable, and implemented consistently across all study participants? | NA | NA | NA | NA | NA | NA | NA | NA | NA | CD | NA | NA | NA | NA | CD |
| 7. Was the length of follow-up adequate? | N | N | N | N | Y | N | Y | Y | Y | CD | Y | N | Y | Y | Y |
| 8. Were the statistical methods well-described? | CD | Y | CD | Y | Y | CD | Y | Y | Y | Y | Y | CD | Y | Y | Y |
| 9. Were the results well-described? | Y | Y | Y | Y | Y | Y | Y | Y | Y | Y | Y | Y | Y | Y | Y |
| Quality rating | Fair | Good | Fair | Good | Good | Fair | Good | Good | Good | Fair | Good | Good | Good | Good | Good |

*CD, cannot determine; NA, not applicable; NR, not reported
